# Non‐Invasive Assessment of Complete Regression in Endometrial Cancer Patients Undergoing Fertility Preservation Using MRI‐Based Radiomics and Immune Heterogeneity

**DOI:** 10.1002/mco2.70666

**Published:** 2026-03-04

**Authors:** Xingchen Li, Kun Shang, Jingyuan Wang, Aoxuan Zhu, Yuman Wu, Yue Qi, Xinyi Bi, Yiqin Wang, Jianliu Wang

**Affiliations:** ^1^ Department of Obstetrics and Gynecology Peking University People's Hospital Beijing China; ^2^ Department of Nuclear Medicine Peking University People's Hospital Beijing China

**Keywords:** endometrial cancer, fertility preservation, immune heterogeneity, prediction, radiomics

## Abstract

Fertility‐preserving treatment (FPT) offers a critical option for young women diagnosed with atypical endometrial hyperplasia (AEH) or early‐stage endometrial cancer (EC), however, the commonly used methods for evaluating complete regression (CR) are invasive. This study aimed to develop a non‐invasive tool to predict treatment outcomes using radiomics and molecular profiling. We retrospectively analyzed 146 patients with AEH or early EC receiving FPT. Radiomic features extracted from MRI were used to construct a radiomics signature predictive of CR through a machine‐learning approach. A radiomics‐clinical nomogram integrating radiomics scores with clinical variables demonstrated excellent predictive performance, with area under the curve values of 0.963 and 0.986 in the training and validation cohorts, respectively. Patients stratified into high‐ and low‐score groups based on radiomics scores showed significantly different CR rates, with the high‐score group exhibiting a lower likelihood of CR. Single‐cell RNA sequencing further confirmed immune alterations in the high‐score group, including reduced CD8^+^ T‐cells, and elevated levels of M2 macrophages. Bulk RNA sequencing revealed upregulation of oxidative phosphorylation and lipid metabolism pathways, suggesting a metabolically active and immunosuppressive tumor microenvironment. This radiomics‐based approach holds promise for guiding individualized FPT strategies for AEH and early EC patients.

## Graphical abstract

Fertility preservation therapy allows young women with early EC to preserve fertility. We developed a non‐invasive MRI radiomics model to predict treatment success. It accurately identifies patients less likely to achieve remission, linking this to an immunosuppressive tumor environment. This aids personalized treatment planning.

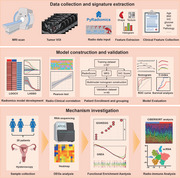

## 1 Introduction

Endometrial cancer (EC) incidence is rising worldwide, particularly in younger women seeking fertility preservation [1]. For early‐stage EC or atypical hyperplasia (AEH), conservative management with progestin is standard [2]. For patients diagnosed with atypical endometrial hyperplasia (AEH) or early‐stage EC who desire future childbearing, conservative management with high‐dose progestin therapy—such as medroxyprogesterone acetate (MPA) or levonorgestrel‐releasing intrauterine device (LNG‐IUD)—has been widely adopted [3]. Although about 70% achieve complete regression (CR), approximately 30% show primary progestin resistance, and recurrence is common [4]. Hysteroscopy is currently the primary method for evaluating the CR status of EC. However, since patients undergoing fertility preservation require reassessment every three months, repeated hysteroscopic procedures can cause endometrial injury and potentially compromise future fertility. Therefore, there is a pressing need to develop a non‐invasive approach for monitoring treatment response.

Radiomics involves the high‐throughput extraction of quantitative features from medical images and has emerged as a non‐invasive tool for characterizing tumors, predicting prognosis, and assessing treatment response [5]. Driven by artificial intelligence and machine learning, it improves predictive accuracy for survival, molecular subtypes, and therapeutic outcomes in various cancers [6]. Radiomic features from T2‐weighted magnetic resonance imaging (MRI) have shown strong potential in reflecting tumor biology, the microenvironment, lymph node metastasis, and fibrosis, surpassing traditional clinical evaluations in many types of cancers [7, 8]. Multimodal integration with histopathology and genomics further enhances risk stratification and personalized treatment planning [9]. These advances highlight radiomics as a promising non‐invasive biomarker, with MRI playing a central role in facilitating precision oncology.

In EC, conventional MRI is essential for preoperative staging, particularly for assessing myometrial invasion, but its performance depends on radiologist experience and suffers from interobserver variability [10]. Radiomics provides a quantitative “virtual biopsy” that captures tumor heterogeneity and improves risk stratification. MRI‐based radiomic models have demonstrated high diagnostic performance in predicting deep myometrial invasion, lymphovascular space invasion (LVSI), and hematologic toxicity risk during radiotherapy. Thus, integrating MRI with radiomics represents a promising strategy for advancing personalized management in EC.

The biological basis of radiomics often lies in its ability to reflect the tumor microenvironment (TME) [11]. Tumors with immunosuppressive TME often display distinct radiological phenotypes, as immune cell infiltration alters tumor structure and heterogeneity [12]. Radiomics, by extracting high‐dimensional features from MRI, offers a non‐invasive approach to capture this biological complexity and predict immune profiles [13]. Radiogenomic analyses have linked radiomic features to immune‐related gene expression signatures, such as CD8+ T‐cell infiltration and PD‐L1 expression [14, 15]. From our perspective, we intend to integrate MRI‐based radiomics with single‐cell transcriptomic profiling to directly analyze immune cell infiltration patterns. This approach allows us to explore the hypothesis that specific radiomic signatures are associated with distinct immune landscapes, providing new insights into tumor‐immune interactions in EC.

In this study, we developed a radiomics‐based predictive model for CR in EC, demonstrating that imaging features significantly enhance predictive accuracy. Furthermore, transcriptomic and single‐cell sequencing analyses revealed that radiomic features reflect immune cell infiltration patterns, linking imaging phenotypes with the underlying tumor immune microenvironment. These findings suggest that radiomics can non‐invasively capture the biological heterogeneity associated with treatment response, offering new insights for precision therapy in EC.

## 2 Results

### 2.1 Clinical Characteristics of Enrolled Patients

**FIGURE 1:**
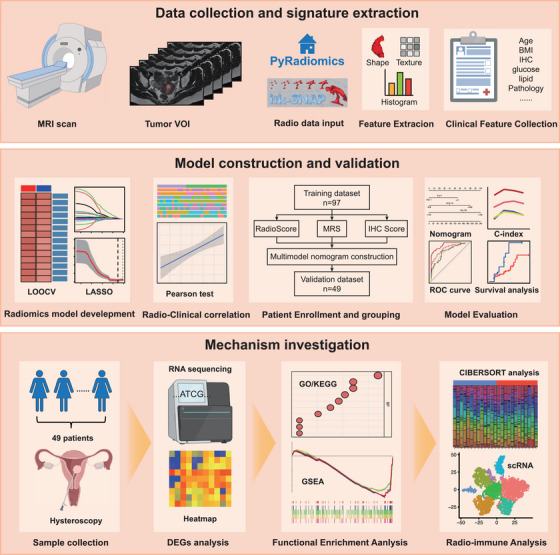
The flow chart of the method. First, ROIs of the primary tumor segmented on T2‐weighted images. Then, these ROIs were applied to three‐dimensionally reconstruct primary tumor. Also, the three‐dimensional radiomics features and deep learning features were extracted for further analysis. Next, based on above features, predictive models were constructed and validated. Finally, RNA sequence and single cell RNA were conducted to illustrate the underlying mechanism.

The flow chart summarizes the study design (Figure 1). A total of 146 patients who met the inclusion criteria were included in this study, with a 2:1 ratio of patients randomly assigned to the training and validation groups. The training group consisted of 97 patients, while the validation group included 49 patients. The two groups were stratified based on CR status. Characteristics of the enrolled patients are presented in Table 1. In the training cohort, significant differences in baseline characteristics were observed according to CR status. The non‐CR group exhibited significantly higher metabolic parameters, including BMI (28.76 ± 4.42 vs. 26.67 ± 5.06 kg/m^2^, *p* = 0.047), fasting blood glucose (6.26 ± 3.15 vs. 4.93 ± 0.79 mmol/L, *p* = 0.012), cholesterol (5.15 ± 0.90 vs. 4.56 ± 0.87 mmol/L, *p* = 0.004), and LDL levels (12.55 ± 48.31 vs. 2.87 ± 0.75 mmol/L, *p* = 0.002) Immunohistochemistry scores were significantly lower in non‐CR patients (*p *< 0.001) No significant age difference was found between other groups (*p* = 0.065) These findings demonstrate distinct clinical profiles between response groups, characterized by adverse metabolic parameters and altered menstrual patterns in non‐CR patients.

**TABLE 1:** Characteristics of patients in this study.

Variables	Training cohort			Validation cohort		
CR status	CR	Non‐CR	*p* value	CR	Non‐CR	*p* value
*N*	69	28		33	16	
Age	32.04 ± 5.00	33.82 ± 4.75	0.065	30.85 ± 4.46	33.56 ± 5.20	0.095
BMI (kg/m^2^)	26.67 ± 5.06	28.76 ± 4.42	0.047	26.05 ± 4.80	27.44 ± 5.77	0.165
Gravida	0.54 ± 0.78	0.25 ± 0.52	0.082	0.48 ± 0.80	0.38 ± 0.62	0.786
Para	0.17 ± 0.42	0.07 ± 0.26	0.246	0.06 ± 0.35	0.19 ± 0.40	0.072
FBG (mmol/L)	4.93 ± 0.79	6.26 ± 3.15	0.012	4.84 ± 0.86	6.68 ± 2.46	<0.001
TG (mmol/L)	1.63 ± 1.09	1.54 ± 0.79	1.000	1.55 ± 1.07	1.97 ± 0.98	0.054
HDL (mmol/L)	1.13 ± 0.40	1.20 ± 0.29	0.094	1.11 ± 0.30	1.09 ± 0.35	0.798
Cholesterol (mmol/L)	4.56 ± 0.87	5.15 ± 0.90	0.004	4.60 ± 0.83	4.74 ± 1.12	0.594
LDL (mmol/L)	2.87 ± 0.75	12.55 ± 48.31	0.002	2.93 ± 0.73	3.02 ± 0.99	0.941
CA125 (U/L)	23.39 ± 23.41	17.51 ± 8.92	0.347	23.55 ± 17.15	18.47 ± 9.46	0.392
ER percentage	0.73 ± 0.23	0.76 ± 0.26	0.285	0.74 ± 0.25	0.82 ± 0.10	0.788
PR percentage	0.79 ± 0.22	0.82 ± 0.20	1.000	0.75 ± 0.25	0.82 ± 0.20	0.546
P53 percentage	0.20 ± 0.34	0.46 ± 0.47	0.022	0.17 ± 0.30	0.18 ± 0.33	0.931
Ki‐67 percentage	0.21 ± 0.13	0.26 ± 0.18	0.290	0.20 ± 0.13	0.24 ± 0.13	0.417
MRS	1.36 ± 3.72	3.14 ± 4.33	0.067	1.00 ± 3.79	5.38 ± 3.72	<0.001
IHC score	0.89 ± 0.08	0.79 ± 0.11	<0.001	0.90 ± 0.08	0.78 ± 0.11	0.002
Radio risk score	125.00 ± 2.00	127.10 ± 1.34	<0.001	124.81 ± 1.73	126.22 ± 1.59	0.007
Hypertension			0.046			0.313
Without	64 (92.75%)	22 (78.57%)		31 (93.94%)	13 (81.25%)	
With	5 (7.25%)	6 (21.43%)		2 (6.06%)	3 (18.75%)	
Diabetes			0.912			0.724
Without	51 (73.91%)	21 (75.00%)		21 (63.64%)	11 (68.75%)	
With	18 (26.09%)	7 (25.00%)		12 (36.36%)	5 (31.25%)	
Menstrual regularity			0.001			0.598
Regular	34 (49.28%)	4 (14.29%)		15 (45.45%)	6 (37.50%)	
Irregular	35 (50.72%)	24 (85.71%)		18 (54.55%)	10 (62.50%)	
PCOS			0.754			0.335
Without	42 (60.87%)	18 (64.29%)		25 (75.76%)	10 (62.50%)	
With	27 (39.13%)	10 (35.71%)		8 (24.24%)	6 (37.50%)	
Pathological type			0.167			0.381
AEH	36 (52.17%)	10 (35.71%)		14 (42.42%)	10 (62.50%)	
EC G1	25 (36.23%)	16 (57.14%)		13 (39.39%)	5 (31.25%)	
EC G2	8 (11.59%)	2 (7.14%)		6 (18.18%)	1 (6.25%)	
Number of ER+			<0.001			0.260
1	42 (66.67%)	9 (33.33%)		19 (63.33%)	6 (46.15%)	
2	10 (15.87%)	2 (7.41%)		5 (16.67%)	1 (7.69%)	
3	11 (17.46%)	16 (59.26%)		6 (20.00%)	6 (46.15%)	
Number of PR+			0.001			0.069
1	36 (57.14%)	7 (25.93%)		19 (63.33%)	4 (30.77%)	
2	11 (17.46%)	2 (7.41%)		5 (16.67%)	2 (15.38%)	
3	16 (25.40%)	18 (66.67%)		6 (20.00%)	7 (53.85%)	
Number of P53+			0.115			1.000
0	22 (44.00%)	6 (25.00%)		11 (50.00%)	5 (50.00%)	
1	28 (56.00%)	18 (75.00%)		11 (50.00%)	5 (50.00%)	

*Note*: CR and non‐CR is divided by 6 months.

Abbreviations: AEH, atypical endometrial hyperplasia; BMI, body mass index; CR, complete regression; EC, endometrial cancer; ER, estrogen receptor; FBG, fasting blood glucose; HDL, high‐density lipoprotein; IHC, immunohistochemistry; LDL, low‐density lipoprotein; MRS, metabolic risk score; PCOS, polycystic ovarian syndrome; PR, progesterone receptor; TG, triglyceride.

### 2.2 Feature Selection and Radiomics Signature Construction

**FIGURE 2:**
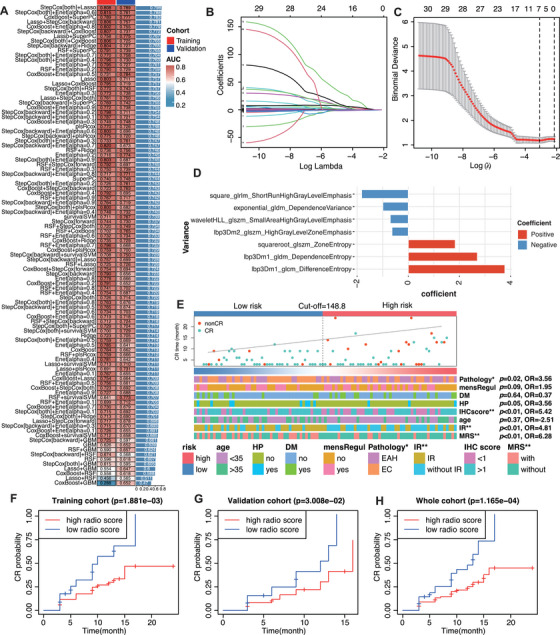
Construction and validation of the radiomics‐related risk model for EC patients. (A) Total of 101 types of prediction models investigated by the Leave‑One‑Out Cross‑Validation (LOOCV) framework. The C‑index of each model was calculated across all validation cohorts. (B) LASSO regression algorithm to screen associated factors with cross‐validation, where factor gene has a different color. (C) The selection with the lowest misclassification error. (D) Coefficients of the 7 factors obtained in LASSO regression. (E) The differences of CR and clinical molecular pathology in patients in different risk groups are shown in heat maps in training cohort. Patients in training cohort are arranged in ascending order of risk score. (F‐H) CR of fertility preservation in (F) training, (G) validation, and (H) whole cohorts.

In the training cohort, a total of 101 prediction models were developed using the LOOCV framework, and the C‐index of each model was calculated (Figure 2A). Stepwise Cox regression (StepCox) and LASSO regression models demonstrated the highest predictive performance, with average C‐index values of 0.808 in the training cohort and 0.789 in the validation cohort, resulting in an overall average C‐index of 0.798. After LASSO shrinkage, seven radiomic features were retained (Figure 2B,C) The corresponding coefficients of the selected features are illustrated in Figure 2D. Using these features and their respective coefficients, a radiomics score (termed CR‐associated radiomics signature, CARS) was calculated for each patient. The median radiomics score (148.8) in the training cohort was used as the cut‐off value for risk subgroup classification in this study. In addition, the distribution of risk scores and patient CR status is shown in the scatter plot (Figure 2E). Validation and whole cohorts indicated similar results (Figure S1A,B). Significant differences were observed in pathology, immunohistochemistry (IHC) score, insulin resistance, and metabolic syndrome between the high‐ and low‐risk groups. The prognostic value of the CARS model was further validated by assessing the CR rates across different risk groups. As shown in Figure 2F–H, patients with a lower radiomics score exhibited significantly higher cumulative CR rates compared with those in the high‐risk group, consistently across the training cohort, validation cohort, and whole cohort. The accuracy of the radiomics‐related model is evaluated by ROC curve. The results showed that the signature has high accuracy in training, with area under the ROC curve (AUC = 0.731, Figure S1C), validation (AUC = 0.744, Figure S1D), and whole (AUC = 0.742, Figure S1E). The cumulative CR curves, which increased over time, indicated that the CARS model effectively stratified patients according to their probability of achieving CR.

### 2.3 Prognostic Value of the Multimodal Nomogram

**FIGURE 3:**
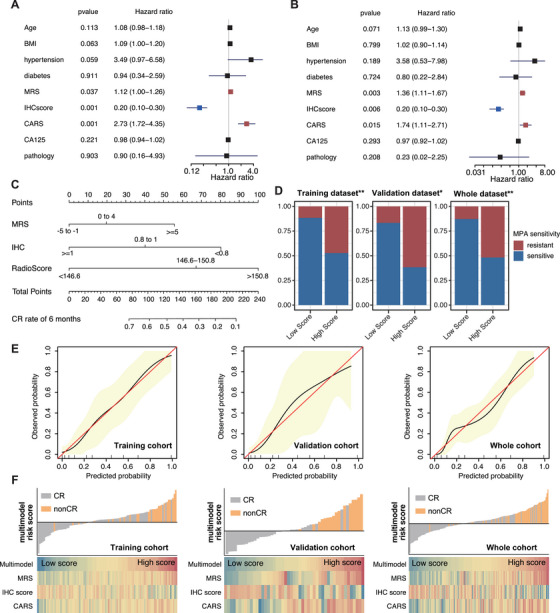
Predictive value of the multimodal nomogram‐defined fertility preservation therapy benefits. (A and B) Univariable and multivariable association of the radiomics signature and clinicopathological characteristics with CR in training cohort. (C) Radiomics‐clinical nomogram developed based on radiomics features and clinicopathological characteristics in training cohort. (D) Distribution of progestin sensitive and resistant patients in low‐ and high‐score groups. (E) Calibration curves of the radiomics‐clinical nomogram in the training, validation, and whole cohort. (F) Waterfall plots demonstrate the incidence of CR events as the multimodal nomogram score increases from left to right, with the high‐score group to the right of the red dashed line showing a significantly lower incidence of CR events compared with the low‐score group on the left. Heatmaps display that the radiomics, IHC score, and metabolic risk score provide complementary prognostic information.

Based on the training cohort, a Cox regression model was employed to evaluate the predictive value of multiple clinicopathological factors, including the metabolic risk score (MRS), IHC score, and CARS. Both univariate and multivariate cox regression analyses (Figure 3A,B) demonstrated that MRS (HR = 1.36, 95% CI: 1.11–1.67, *p* = 0.003), IHC score (HR = 0.20, 95% CI: 0.10–0.30, *p* = 0.006), and CARS (HR = 1.74, 95% CI: 1.11–2.71, *p* = 0.015) were independent predictors of achieving CR. A multimodal nomogram integrating these three independent factors was then constructed to predict the probability of CR in fertility‐preserving patients with EC (Figure 3C). Using the multimodal nomogram, patients were classified into high‐ and low‐score groups according to the optimal cut‐off value. Correlation analysis revealed that the distribution of progestin resistance differed significantly between the high‐ and low‐ score groups across the training, validation, and whole cohorts, with a higher proportion of progestin‐resistant patients observed in the high‐score group (Figure 3D). Calibration curves for the nomogram in the three cohorts (Figure 3E) showed good agreement between predicted and observed probabilities of achieving CR, indicating high accuracy. Moreover, the heatmap illustrates the distribution of CR outcomes between the low‐ and high‐score groups defined by the multimodal nomogram, showing that patients in the high‐ score group were less likely to achieve CR compared with those in the low‐score group, demonstrating the robust stratification ability of the multimodal nomogram (Figure 3F). Overall, the multimodal nomogram integrating the MRS, IHC score, and CARS was developed and validated as an effective predictor for CR in EC patients undergoing fertility‐preserving treatment.

### 2.4 Prognostic Value of the Multimodal Nomogram

**FIGURE 4:**
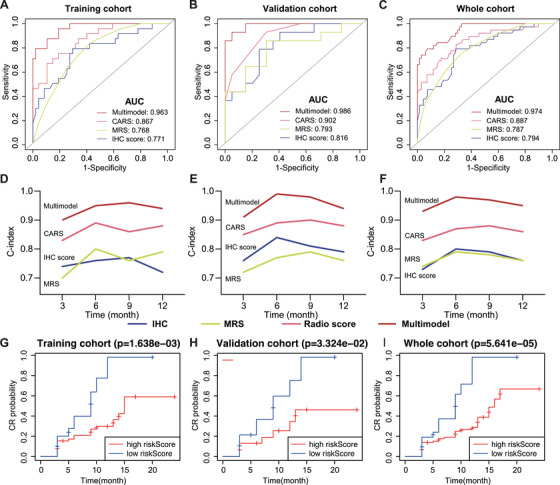
Evaluation of the multimodal nomogram in different patient cohorts. Area under the time‐dependent ROC curves for predicting CR using the unimodal scores and the multimodal nomogram in the training (A), validation (B), and whole cohorts (C). (D–F) The C‐index for CR prediction using the multimodal nomogram predicting 3, 6, 9, and 12‐month CR. (G–I) CR curves show that the multimodal nomogram‐defined high/low‐score groups have promising stratification ability across different cohorts.

The predictive efficacy of the multimodal nomogram was evaluated in the training, validation, and whole cohorts. As shown in Figure 4A–C, in the training cohort, the AUC for predicting CR was 0.771 for the IHC score, 0.768 for the MRS, and 0.867 for the CARS, respectively. When combined into a multimodal model, the AUC increased markedly to 0.963. In the validation cohort, the AUCs were 0.816 (IHC score), 0.793 (MRS), and 0.902 (CARS), with the multimodal model achieving an AUC of 0.986. Similarly, in the whole cohort, the AUCs were 0.794, 0.787, and 0.887 for the three factors respectively, while the multimodal model reached an AUC of 0.974. The time‐dependent C‐index for predicting 3‐, 6‐, 9‐, and 12‐month CR was consistently higher for the multimodal nomogram compared to any individual predictor across all three cohorts (Figure 4D–F), highlighting the robust and stable performance of the multimodal approach over time. Furthermore, Kaplan–Meier (KM) curves for CR stratified by the nomogram‐derived risk scores were plotted (Figure 4G–I). In all three cohorts, patients in the high‐score group exhibited significantly lower CR rates, while those in the low‐score group achieved markedly higher CR rates. These findings confirmed the nomogram's excellent ability to stratify patients based on the likelihood of achieving CR. The predictive performance of the MRS, IHC score, CARS, and multimodal models was compared across the training, validation, and whole cohorts (Table 2). The multimodal model consistently demonstrated superior performance compared to the other models in all cohorts, with an AUC of 0.96 (95% CI: 0.93–0.99) in the training set, 0.98 (95% CI: 0.95–1.00) in the validation set, and 0.97 (95% CI: 0.95–0.99) in the whole cohort. These values were significantly higher than those of the MRS, IHC, and CARS models (all *p *< 0.001) Overall, the multimodal model demonstrated significantly better predictive accuracy, with consistently higher AUCs. This highlights its strong generalizability and superior ability to predict outcomes compared to the MRS, IHC, and CARS models.

**TABLE 2:** Performance of models for predicting the CR status.

	AUC (95%CI)	Sensitive	Specificity	Accuracy	*p* value
MRS					
Training	0.76 (0.65–0.87)	0.83 [42/51]	0.58 [27/46]	0.67 [69/97]	<0.001
Validation	0.79 (0.63–0.95)	0.86 [25/29]	0.70 [14/20]	0.77 [39/49]	0.024
Whole	0.78 (0.70–0.87)	0.74 [59/80]	0.69 [46/66]	0.70 [105/146]	<0.001
IHC score					
Training	0.77 (0.65–0.89)	0.80 [41/51]	0.72 [33/46]	0.76 [74/97]	<0.001
Validation	0.81 (0.67–0.95)	0.79 [23/29]	0.75 [15/20]	0.78 [38/49]	0.015
Whole	0.79 (0.69–0.88)	0.80 [64/80]	0.73 [48/66]	0.77 [112/146]	<0.001
CARS					
Training	0.86 (0.78–0.95)	0.77 [39/51]	0.83 [38/46]	0.79 [77/97]	0.012
Validation	0.90 (0.80–0.99)	0.93 [27/29]	0.70 [14/20]	0.83 [41/49]	0.035
Whole	0.88 (0.74–0.96)	0.87 [66/80]	0.79 [52/66]	0.80 [118/146]	0.004
Multi‐model					
Training	0.96 (0.93–0.99)	0.96 [49/51]	0.83 [38/46]	0.90 [87/97]	ref
Validation	0.98 (0.95–1.00)	0.93 [27/29]	0.95 [19/20]	0.94 [46/49]	ref
Whole	0.97 (0.95–0.99)	0.95 [76/80]	0.86 [57/66]	0.91 [/146]	ref

Abbreviations: AUC, area under the curve; CARS, CR‐associated radiomics signature; CR, complete regression; IHC, immunohistochemistry; MRS, metabolic risk score.

### 2.5 Transcriptomic Analysis Between High‐ and Low‐Score Groups of Fertility‐Preserving Endometrial Cancer Patients

**FIGURE 5:**
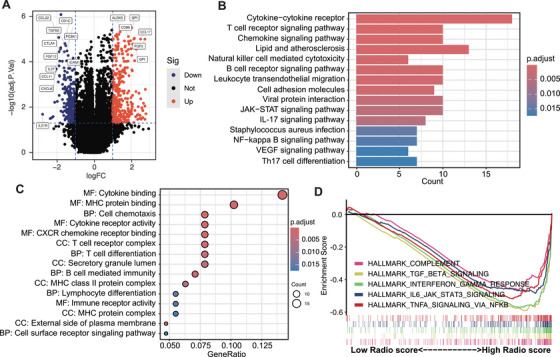
Biological patterns via transcriptome analysis of radiomics‐related signature. (A) Volcano plot shows the differentially expressed genes between radiomics model‐predicted low‐ and high‐score groups. (B) Bar plots show the top 15 representative correlated pathways with the radiomics model score based on the gene set variation analysis scores of KEGG pathway. (C) The bubble plot shows the result of GO analysis from the Gene Ontology Biological Function database. (D) Gene set enrichment analysis shows the pathways associated with the radiomics‐related differentially expressed genes from the result of hallmark pathway analysis.

To further explore the molecular mechanisms underlying the risk classification, we performed transcriptomic sequencing of 49 EC fertility‐preserving patients. Based on the cutoff values, patients were categorized into high‐ and low‐score groups, and differential gene expression analysis was conducted to identify key genes associated with prognosis. The volcano plot in Figure 5A illustrates the upregulated and downregulated genes between the two groups. A total of 104 upregulated genes and 152 downregulated genes were identified, highlighting significant molecular differences between the groups. KEGG pathway analysis revealed that differentially expressed genes were enriched in several key pathways, including the cytokine‐cytokine receptor interaction, T cell receptor signaling pathway, and natural killer cell‐mediated cytotoxicity, which are critical for immune response and tumor progression (Figure 5B). These pathways suggest an involvement of immune system modulation in the high‐score group, potentially contributing to worse prognosis. GO analysis further emphasized functional differences, with significant enrichment in processes such as cytokine binding, T cell receptor complex formation, and lymphocyte differentiation (Figure 5C), all of which are closely related to immune responses. Gene set enrichment analysis (GSEA) in Figure 5D highlighted significant enrichments in immune and inflammatory response pathways, such as interferon‐gamma response, IL6_JAK_STAT3 signaling, and TNF‐α_signaling via NFκB, specifically in the high‐score group. These findings support the notion that immune‐related pathways, including inflammation and cytokine signaling, may play pivotal roles in the progression of EC and its response to treatment [16]. Overall, the transcriptomic data from this analysis provide valuable insights into the molecular characteristics of high‐ and low‐score groups, with immune‐related pathways emerging as key factors influencing prognosis in fertility‐preserving patients.

### 2.6 Immune Cell Infiltration and Validation of Radiomic Risk Model in EC

**FIGURE 6:**
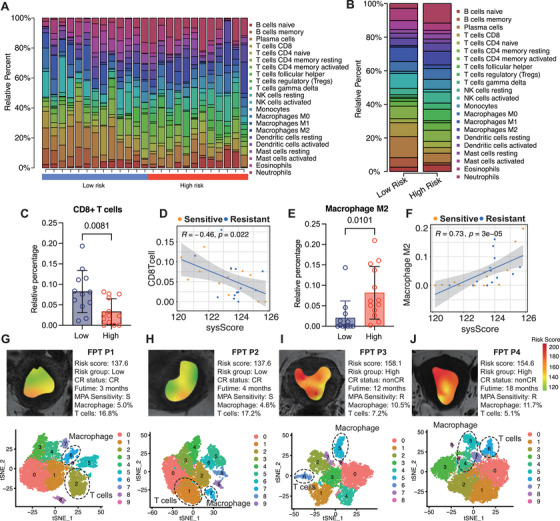
Immune characteristics of radiomics‐related to high‐ and low‐score groups. (A and B) The fraction of the multiple cell types indicated proportional diversity between high‐ and low‐score groups via the CIBERSORT algorithm. (C) Compare the differences in the proportion of CD8+ T cells between the high‐ and low‐score groups. (D) Correlative analysis between radiomics risk score and proportion of CD8+ T cell. (E) Compare the differences in the proportion of M2 type macrophage cells between the high‐ and low‐score groups. (F) Correlative analysis between radiomics risk score and proportion of M2 type macrophage cell. (G and J) Representative MRI images of patients in high‐ and low‐score groups. In the radiomics maps feature warmer and cooler colors indicate higher and lower risk scores, respectively. tSNE plot shows clustering of each patient's cells based on gene expression. Point coordinates are based on tSNE dimensionality reduction of the top principal components calculated from the 5000 most informative genes. Cell color specifies assignment of cells to these clusters inferred using shared nearest neighbor clustering. T cell and macrophage are marked. (G and H) Two patients in low score group. (I and J) Two patients in high score group.

In this study, we compared immune cell infiltration between the high‐ and low‐score groups based on radiomic score to further investigate the tumor immune microenvironment. Figure 6A shows a heatmap of the 22 immune cell types estimated from the transcriptomic data of the fertility‐preserving EC patients using Cell‐type Identification By Estimating Relative Subsets Of RNA Transcripts (CIBERSORT). We observed significant differences in immune cell profiles between the two groups (Figure 6B). Notably, the low‐score group exhibited a higher infiltration of CD8+ T cells (*p* < 0.05; Figure 6C,D), consistent with the upregulation of immune‐related pathways, particularly the interferon (IFN)‐related pathways. On the other hand, M2 macrophages were more abundant in the high‐score group (*p *< 0.05; Figure 6E), with their levels showing a positive correlation with the radiomic risk score (Figure 6F). What is more, the distribution of NK cell, macrophage M1, neutrophil, and Treg also diverse between the two risk groups (Figure S2). To further validate these results, we performed single‐cell RNA sequencing (scRNA‐seq) on tissue samples from four patients with varying risk scores (detailed cell marker, see Table S1). Patient 1, with a low‐score score of 137.8, achieved CR after three months of progestin therapy, showing a relatively low overall risk score in the radiomic heatmap (Figure 6G). Single‐cell analysis revealed that M2 macrophages comprised only 5.0% of the cells in this patient (Cluster 6), while T cells accounted for 16.8% (Cluster 2) Patient 2 also exhibits similar result (Figure 6H). In contrast, Patient 3, with a high‐score of 158.1, did not reach CR after 12 months and exhibited significantly higher M2 macrophage content (10.5%) and a lower proportion of T cells (7.2%) (Figure 6I). Another high‐score shows a similar result (Figure 6J). These findings were consistent with the radiomic model, where high‐score patients demonstrated increased M2 macrophage infiltration and reduced T cell presence. In summary, the comparison of immune cell infiltration between the high‐ and low‐score groups, along with the validation using scRNA‐seq data from individual patients, supports the conclusion that the low‐score group is associated with higher levels of CD8+ T cells and a more favorable immune response, whereas the high‐score group shows elevated M2 macrophage levels. These results reinforce the potential utility of radiomic signatures in predicting immune cell infiltration and treatment responses in EC.

## 3 Discussion

In this study, we developed and validated CARS, a radiomics model based on multi‐parametric MRI, to predict complete regression in EC patients seeking fertility preservation. The model demonstrated robust performance in internal and overall validation. We further built a clinically applicable nomogram by integrating the CARS score with IHC markers and an MRS, which significantly improved predictive accuracy for early intervention. Mechanistically, the model's predictions were linked to a distinct immunosuppressive tumor microenvironment, characterized by reduced CD8^+^ T‐cell infiltration and increased M2 macrophage presence, as confirmed by single‐cell transcriptomics. Our findings position radiomics as a non‐invasive tool for early outcome prediction and provide insights into the biology of therapeutic effects.

Radiomics has become a valuable non‐invasive tool for tumor characterization in endometrial cancer (EC). MRI‐based radiomics can predict key prognostic factors like deep myometrial invasion, LVSI, and molecular features, often achieving AUCs >0.9 [17, 18]. Integrating deep learning, clinical variables, and peritumoral regions further improves molecular subtyping and prediction of biomarkers such as TP53 status [19]. Despite scanner variability, multi‐institutional studies confirm its robustness and generalizability [17, 20]. As a cost‐effective and reproducible supplement to histology, radiomics supports personalized, preoperative decision‐making in EC [21]. Our study extends these advancements by specifically developing and validating a multimodal radiomics‐based nomogram that integrates MRS, IHC score, and CARS for predicting complete regression—a critical yet underexplored clinical challenge in fertility‐preserving management. The achieved superior predictive performance not only aligns with the high accuracy reported for other EC biomarkers but also demonstrates the added value of an integrated model in addressing therapeutic response prediction beyond traditional pathological staging.

In recent years, machine learning and AI have significantly advanced radiomics‐based tumor prediction. Deep learning techniques such as convolutional neural networks and feature fusion have improved the accuracy and efficiency of medical image analysis. For instance, one study achieved 84.6% sensitivity and 75% specificity in EC risk prediction using fluorescence lifetime imaging microscopy [22]. While another approach automatically detected EC from hysteroscopic images with nearly 90% accuracy [23]. Similarly, deep learning frameworks have been introduced to analyze multi‐sequence MRI, enabling automated tumor segmentation and prediction of lymph node metastasis and LVSI [24]. These developments highlight the ability of AI to model complex tumor behavior, with growing interpretability through feature visualization and linkage to immune and molecular data.

Radiomics has further shown promise in early‐stage EC assessment. Models using logistic regression can distinguish stage IA EC from benign lesions [25], while other approaches detect occult EC and assess myometrial invasion [26]. Integration of radiomic and clinical data has improved the prediction of deep myometrial invasion, with texture and first‐order features capturing tumor heterogeneity beyond human perception [27]. However, many earlier models focused only on the tumor core, neglecting the peritumoral microenvironment. Recent evidence indicates that including peritumoral features—particularly within a 5 mm margin—can enhance performance by reflecting stromal and immune activity [28]. Moreover, multimodal radiomics models that combine T2WI, DWI, and DCE‐MRI sequences consistently outperform unimodal ones, likely due to the complementary biological insights each sequence provides [29]. Recent efforts have also aimed to predict molecular subtypes such as MMRd and p53abn using radiomic features and clinical data, achieving moderate AUC values [30]. To address these gaps, our model integrates radiomics feature with clinical variables, providing a more robust framework for individualized risk stratification.

The clinical and translational significance of our model lies in its potential to refine personalized treatment strategies for women undergoing fertility‐preserving management. By providing a non‐invasive and quantitative assessment of the likelihood of CR, the model could aid clinicians in identifying patients who are optimal candidates for progestin therapy versus those at high risk of resistance, for whom closer surveillance or upfront alternative interventions might be considered. Integration into current diagnostic workflows is feasible, as it builds upon routine preoperative MRI, requiring only an additional, automated post‐processing step to generate the radiomics signature. Future efforts should focus on developing a user‐friendly software interface for seamless feature extraction and model calculation in a clinical setting, followed by prospective multi‐center trials to validate its efficacy in real‐time decision‐making and ultimately improve oncologic and reproductive outcomes.

Our study identified that radiomic features are associated with immune cell infiltration, particularly CD8^+^ T‐cells and macrophages, in EC. Through GO, KEGG, and GSEA analysis, we found significant enrichment in pathways such as cytokine‐cytokine receptor interaction, T cell receptor signaling, and chemokine signaling. These pathways suggest that immune modulation plays a key role in the prediction of CR. Furthermore, the molecular function of cytokine binding was enriched, highlighting the importance of the cytokine network within the TME and its potential influence on immune escape [31]. We also observed upregulation of JAK‐STAT3 signaling and TGF‐β pathway, which are linked to immune suppression, epithelial‐mesenchymal transition (EMT), and progestin resistance [32]. Notably, our investigation also revealed a significant association between the MRS and the observed transcriptomic and immune landscape. Metabolic reprogramming is a recognized hallmark of cancer, and in the context of our study, alterations in pathways such as glycolysis and oxidative phosphorylation—implicit in the MRS—may create an immunosuppressive TME [33]. For instance, a high MRS, indicative of a hypermetabolic state, could lead to nutrient competition and accumulation of immunosuppressive metabolites, thereby inhibiting the function of cytotoxic CD8^+^ T‐cells and promoting the recruitment and polarization of pro‐tumorigenic macrophages [34]. This metabolic‐immune crosstalk potentially explains the concurrent enrichment of immunosuppressive signaling pathways and the specific immune cell infiltration patterns associated with complete regression [35]. Furthermore, this biological exploration uniquely shifts to single‐cell analysis of the immune microenvironment, offering novel insights into the mechanisms of treatment response.

Unlike our previous model predicting progestin resistance in this EC cohort, this study focuses on predicting complete regression [36]. Key distinctions include a different patient partition, an extensive screening of 101 machine learning algorithms, the development of a clinical‐radiomic nomogram, and a unique biological validation through single‐cell analysis of the immune landscape, collectively providing a novel and complementary predictive tool.

This study has several limitations. First and foremost, its single‐center retrospective design is a primary constraint, which may introduce selection bias and limit the model's generalizability to broader populations. The inherent risk of overfitting associated with such a design is acknowledged, and future multi‐center, prospective studies are imperative to validate and refine our model. Second, while the current study focuses on specific radiomic features, future research should incorporate additional imaging sequences to optimize the model further. Finally, the mechanisms through which immune cell infiltration is mapped to radiomic features remain unclear and warrant further exploration to better understand the underlying biological processes.

## 4 Conclusion

This study developed an interpretable, multiparametric MRI‐based radiomics model to predict complete regression in EC. By integrating radiomics features, the model non‐invasively stratifies risk and provides insights into the tumor immune microenvironment. Our findings underscore radiomics' potential to bridge imaging phenotypes with biological processes, aiding preoperative precision medicine and personalized treatment decisions for EC patients undergoing fertility‐preservation treatment.

## 5 Materials and Method

### 5.1 Patient Enrollment

We retrospectively analyzed data from patients who underwent fertility‐sparing therapy for AEH and early‐stage EC (grade 1 or grade 2, EC G1/G2) at Peking University People's Hospital (PKUPH) between January 2012 and January 2025. Baseline clinical characteristics, pathological findings, and follow‐up data were collected. Patients were eligible for inclusion if they met the following criteria: (1) aged 45 years or younger; (2) had a strong desire for fertility preservation and provided informed consent for fertility‐sparing treatment; (3) underwent diagnostic hysteroscopy for endometrial tissue sampling; (4) had no contraindications to progestin therapy; and (5) had available pre‐treatment T2‐weighted MRI imaging data. Preoperative MRI data were retrospectively retrieved from the imaging archive of PKUPH. All patients provided written informed consent for treatment and for the use of their clinical data in health research. Patients were randomly assigned to either a training cohort or a validation cohort at a 2:1 ratio. This study was conducted in accordance with the principles of the Declaration of Helsinki and was approved by the Ethics Committee of Peking University People's Hospital (Approval No. 2022PHB379‐001).

### 5.2 Radiomic Features Extraction

The tumor region of interest (ROI) was manually delineated on contrast‐enhanced T2‐weighted MR images, as this sequence provides excellent soft tissue contrast for endometrial lesions [37]. Eligible patients from the PKUPH center were included for analysis. ROI segmentation was performed independently by two radiologists, each with more than 10 years of experience in gynecological imaging, using the ITK‐SNAP software (version 3.8.0, www.itksnap.org) [38]. The ROI was carefully outlined across all slices to encompass the entire tumor volume while avoiding cystic, necrotic, or hemorrhagic areas. Prior to feature extraction, all MR images underwent a standardized preprocessing pipeline to ensure data consistency and minimize the impact of inter‐scanner variability. This included (1) image resampling to a uniform voxel size of 1 × 1 × 1 mm^3^ to standardize spatial resolution; (2) intensity discretization using a fixed bin width of 25 to reduce noise and normalize intensity distributions across patients; and (3) intensity normalization by *Z*‐score transformation based on the mean and standard deviation of the entire ROI to mitigate scanner‐specific calibration differences. For each patient, a total of 1731 radiomic features were extracted from the delineated ROIs using the open‐source PyRadiomics package (https://pyradiomics.readthedocs.io). The extraction was configured to utilize the preprocessed images and the specified discretization parameters to ensure feature reproducibility. The extracted features were categorized into four groups: (i) first‐order statistics (*n* = 18); (ii) shape and size features (*n* = 13); (iii) textural features derived from gray‐level matrices including the gray‐level co‐occurrence matrix, gray‐level run length matrix, gray‐level size zone matrix, and gray‐level dependence matrix (*n* = 68); and (iv) filter‐derived features, including wavelet (*n* = 688), Laplacian of Gaussian (LoG, *n* = 258), local binary patterns (LBP, *n* = 258), and other filter transformations such as square, square root, logarithm, exponential, and gradient (*n* = 430) [39]. The detailed calculation formulas for each radiomic feature are available on the official PyRadiomics website. All radiologists were blinded to the clinical outcomes during the delineation process to minimize bias.

### 5.3 Radiomics Feature Selection and Risk Stratification

Our modeling process began with the development and comparison of 101 prediction models within the LOOCV framework. After selecting candidate features through leave‐one‐out cross‐validation (LOOCV), the least absolute shrinkage and selection operator (LASSO) logistic regression was further applied to identify the most important predictors associated with CR. The optimal penalty parameter (λ) for the LASSO regression was determined through 10‐fold cross‐validation using the minimum criterion, which led to the selection of seven radiomic features. Risk scores for each patient were calculated by summing the products of each selected feature's value and its corresponding LASSO‐derived coefficient. The specific calculation formula was: Risk Score = (feature1 × coefficient1) + (feature2 × coefficient2) + … + (feature7 × coefficient7). Based on the median value of the risk scores, patients were stratified into high‐risk and low‐risk groups. The distribution of clinicopathological characteristics and the CR curves were compared between the two groups. Prior to LASSO regression, all features were *z*‐score normalized to standardize the data. Features with high interobserver reproducibility (intraclass correlation coefficient, ICC > 0.75) were retained, and in cases of high collinearity (Pearson correlation coefficient > 0.9), the feature with greater clinical interpretability and stability was preserved. LASSO regression was performed with penalty parameters determined by cross‐validation, and features with nonzero coefficients were considered significant predictors. Model performance was comprehensively evaluated using KM survival analysis, aiming to establish a robust and clinically applicable predictive model for CR in patients undergoing fertility‐sparing treatment. To ensure a consistent and biologically relevant approach for radiomic feature extraction, a standardized ROI segmentation protocol was employed for all patients. For cases with a clearly delineated lesion on T2‐weighted MRI, the ROI was carefully traced along the entire tumor boundary. In instances where no discrete lesion was identifiable (e.g., due to prior biopsy or MPA treatment‐induced endometrial atrophy), the ROI was delineated to encompass the entire endometrial cavity as visualized on the T2‐weighted sequence.

### 5.4 Construction of the Radiomics‐Clinical Nomogram

To further improve predictive performance, we integrated radiomic risk scores with clinical and pathological variables to construct a comprehensive nomogram. First, clinical factors with significant differences identified by univariate logistic regression analysis were entered into a multivariate logistic regression model to determine independent predictors associated with CR. The radiomics risk score, together with the independent clinical predictors, was used to build a radiomics–clinical nomogram. Model construction was conducted separately in the training cohort, and the resulting model was subsequently applied to the validation cohort and the whole cohort (training plus validation) for assessment. The calibration of the nomogram was evaluated by calibration curves in all three cohorts, examining the consistency between predicted and observed outcomes. Additionally, we analyzed the distribution of clinical factors across different risk score groups (from high to low), as stratified by the nomogram, to verify the discriminative capacity of the model.

### 5.5 RNA Sequencing

Fresh‐frozen tumor samples were collected from 49 patients undergoing fertility‐preserving treatment and stored immediately in liquid nitrogen after surgery. Total RNA was extracted from these samples, and transcriptome sequencing was performed using the Illumina NovaSeq 6000 platform, following the manufacturer's standard protocols. The pathological diagnosis of all tumor specimens was independently verified by two experienced pathologists. RNA quality and integrity were assessed using an Agilent 2100 Bioanalyzer before sequencing. All sequencing procedures were conducted at the Molecular Pathology Testing Center.

### 5.6 Differential Gene Expression Analysis

Based on the radiomics scores, patients were divided into high‐ and low‐score groups. Raw read counts from RNA sequencing were normalized and analyzed using the “DESeq2” R package (v1.38.3) to identify differentially expressed genes (DEGs) between the two groups. The selection criteria for DEGs were set as |log_2_ fold change (FC)| > 1 and an adjusted *p* value < 0.05 using the Benjamini–Hochberg correction method. The resulting DEGs were visualized using volcano plots and heatmaps. These DEGs provided the basis for downstream functional enrichment analyses.

### 5.7 Functional Enrichment and Pathway Analysis

To elucidate the biological differences associated with radiomics‐defined risk groups, functional enrichment analysis was performed. Gene Ontology (GO) biological processes and Kyoto Encyclopedia of Genes and Genomes (KEGG) pathways were analyzed using the “clusterProfiler” R package (v4.6.2) GSEA was conducted based on the ranked gene list ordered by log_2_FC values, utilizing gene sets from the Molecular Signatures Database (MSigDB v2023.1), including Hallmark, GO, and KEGG collections. Pathways with a normalized enrichment score (|NES| > 1) and adjusted *p* value < 0.05 were considered significantly enriched. Additionally, Gene Set Variation Analysis (GSVA) was performed to calculate enrichment scores of immune‐related signatures for each sample, enabling further comparison of immune pathway activation between groups [40].

### 5.8 Tumor Immune Microenvironment Analysis

To investigate the tumor immune microenvironment differences between radiomics‐defined risk groups, we estimated immune cell infiltration in the 49 tumor samples using the CIBERSORT algorithm. A validated leukocyte gene signature matrix (LM22), which includes 22 immune cell types, was utilized as a reference [41]. The relative proportions of immune cells in each sample were calculated based on normalized RNA sequencing data. Samples with a CIBERSORT output p‐value < 0.05 were included for further analysis. Subsequently, we compared the estimated immune cell proportions between the high‐ and low‐score groups using an unpaired two‐tailed Student's *t*‐test. Additionally, Pearson correlation analysis was performed to assess the association between radiomic signature scores and the abundance of each immune cell type across all samples. Immune cell types showing significant correlations (*p* < 0.05) were identified for further interpretation regarding their potential role in treatment and disease progression.

### 5.9 Risk Heatmap Overlay

Risk score maps were generated by applying the trained radiomic model voxel‐wise across the MRI images. Each voxel was assigned a risk score based on extracted radiomic features. The resulting risk scores were normalized and mapped to a color scale using the jet colormap. The heatmaps were then overlaid onto the corresponding MRI images with partial transparency to visualize the spatial heterogeneity of risk. All processing was conducted using Python (v3.8) with the SimpleITK, NumPy, and Matplotlib libraries.

### 5.10 Single‐Cell Sequencing, Data Processing, and Annotation

Tissues were dissociated into single‐cell suspensions using enzymatic digestion (collagenase I/IV and DNase I), followed by filtration and red blood cell lysis. Cell viability was assessed, and dead cells were removed. Single‐cell libraries were prepared using the 10x Genomics Chromium Controller (Single Cell 3’ v3 Reagent Kits), targeting 3000–8000 cells per sample. Libraries were sequenced on Illumina NovaSeq 6000 platforms. Raw data were processed with CellRanger (GRCh38 reference genome), and quality control in Seurat excluded low‐quality cells. Doublets (7.6%) were removed via DoubletFinder. Data integration was performed using Seurat's “FindIntegrationAnchors” and Harmony. Dimensionality reduction was done with PCA and UMAP. Unsupervised clustering revealed distinct cell populations, which were annotated based on markers: macrophages and T cells. Differential gene expression was analyzed with the Wilcoxon rank‐sum test, and functional enrichment analysis was performed using Metascape to explore cell‐type‐specific biological pathways.

### 5.11 Statistical Analysis

All statistical analyses were performed using R software (v4.2.2), SPSS (v26.0, IBM Corp.), and Python (v3.8). Differences between categorical variables were compared using the *χ* ^2^ test or Fisher's exact test, and differences between continuous variables were assessed using the Mann–Whitney *U* test or Student's *t*‐test as appropriate. A two‐sided *p* value < 0.05 was considered statistically significant.

## Author Contributions

X.C. Li and K. Shang conceived and supervised the project., A.X. Zhu and J.Y. Wang designed and performed the research. K. Shang and X.Y. Bi collected the EC samples and cases. X.C. Li, J.Y. Wang, and Y.Q. Wang performed the data analyses. X.Y. Bi, Y.M. Wu, and X.C. Li and K. Shang performed experiments. Y. Qi, Y.Q. Wang, and J.L. Wang interpreted the results. X.C. Li wrote original manuscript. J.L. Wang reviewed and revised the manuscript. All authors have read and approved the final manuscript.

## Funding

This study is supported by the Noncommunicable Chronic Diseases‐National Science and Technology Major Project (2023ZD0512100), Major Projects of National Science and Technology (2025ZD0545900 and 2025ZD0545901), and Peking University Clinical Scientist Training Program and the Fundamental Research Funds for the Central Universities (BMU2025PYJH007).

## Ethics Statement

All methods in our study were carried out in accordance with the Declaration of Helsinki. The authors are accountable for all aspects of the work in ensuring that questions related to the accuracy or integrity of any part of the work are appropriately investigated and resolved. The study was approved by Institutional Review Board (Approval number: 2022PHB379‐001) in Peking University People's Hospital.

## Consent

Informed consent was obtained from all patients.

## Conflicts of Interest

The authors declare no conflicts of interest.

## Supporting information




**Figure S1**: Receiver operator characteristic curves of the radiomics signature in the (A) training, (B) validation, and (C) whole cohorts. The differences of complete regression and clinical molecular pathology in patients in different risk groups are shown in heat maps in (D) validation and (E) whole cohorts.
**Figure S2**: Compare the differences and correlative analysis in the proportion of different cells between the high‐ and low‐risk groups in (A) NK cell, (B) macrophage M1 cell, (C) Neutrophils, (D) Treg.

**Table S1**: Cell marker for scRNA clustering.

## Data Availability

Data from this study, including de‐identified participant data, will be made available to others upon reasonable request. Additional documents, including the study protocol, statistical analysis plan, and informed consent form, are also available. Data are available from the corresponding author (wangjianliu@pkuph.edu.cn) upon reasonable request.
