# Supplementary material for: Non‐Invasive Assessment of Complete Regression in Endometrial Cancer Patients Undergoing Fertility Preservation Using MRI‐Based Radiomics and Immune Heterogeneity
Source: MedComm (2020). 2026 Mar 4;7(3):e70666. doi: 10.1002/mco2.70666 (PMC12960057; doi:10.1002/mco2.70666)
Supplement: Supplementary file 1 — Figure S1: Receiver operator characteristic curves of the radiomics signature in the (A) training, (B) validation, and (C) whole cohorts. The differences of complete regression and clinical molecular pathology in patients in different risk groups are shown in heat maps in (D) validation and (E) whole cohorts. Figure S2: Compare the differences and correlative analysis in the proportion of different cells between the high‐ and low‐risk groups in (A) NK cell, (B) macrophage M1 cell, (C) Neutrophils, (D) Treg. [file MCO2-7-e70666-s001.docx]

Supplementary data


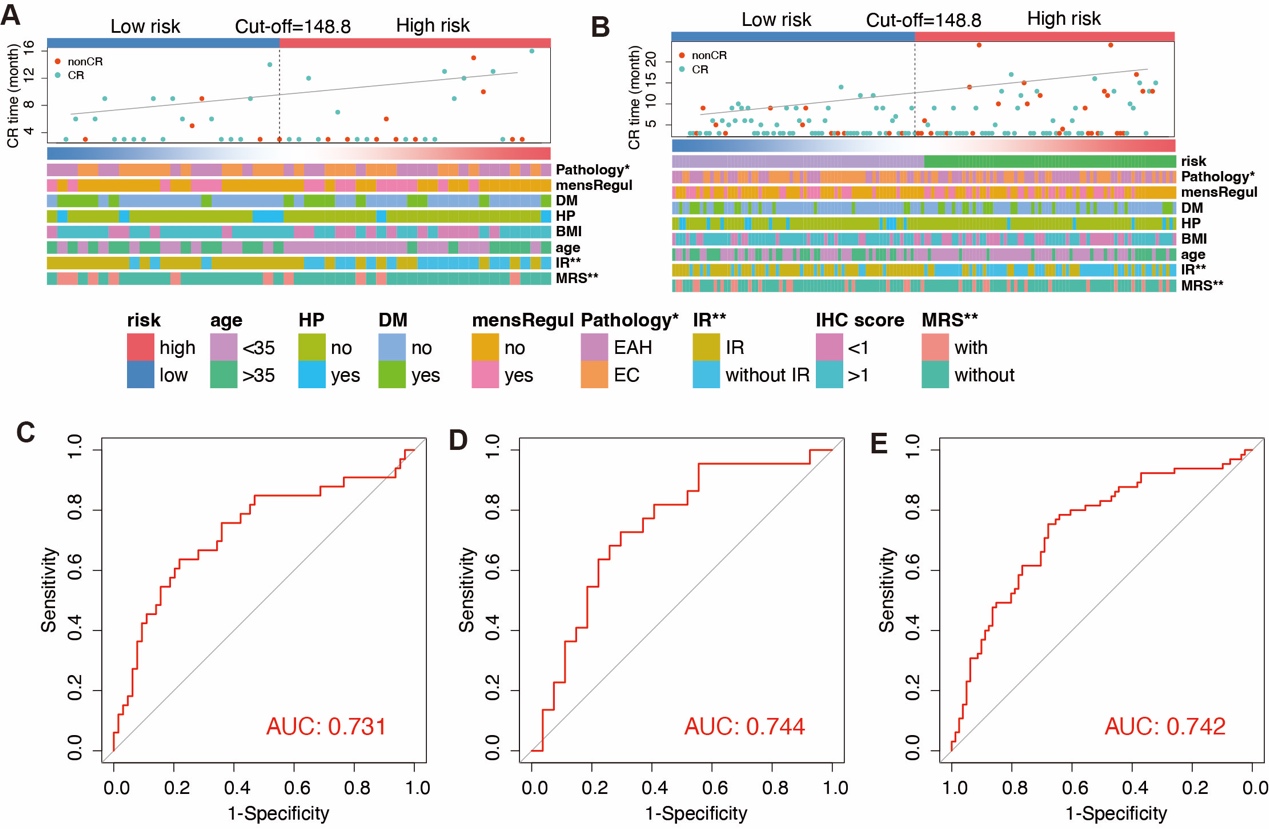


Figure S1. Receiver operator characteristic curves of the radiomics signature in the (A) training, (B) validation, and (C) whole cohorts. The differences of complete regression and clinical molecular pathology in patients in different risk groups are shown in heat maps in (D) validation and (E) whole cohorts.


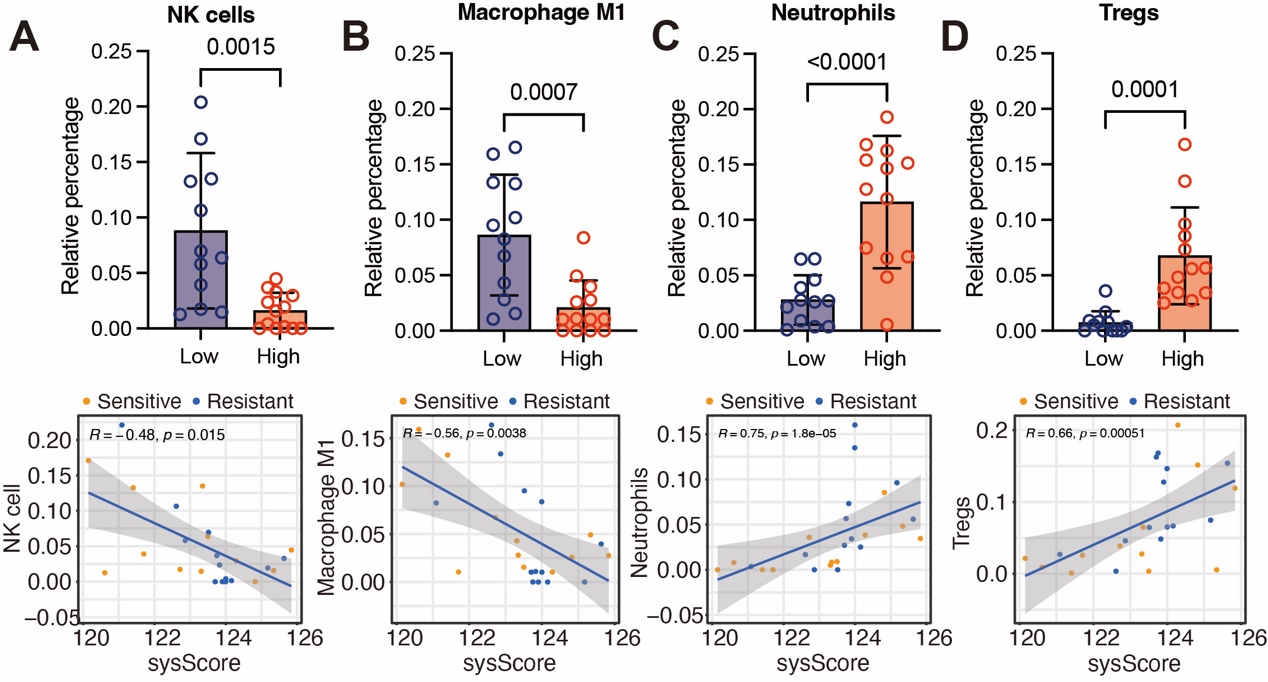


Figure S2. Compare the differences and correlative analysis in the proportion of different cells between the high- and low-risk groups in (A) NK cell, (B) macrophage M1 cell, (C) Neutrophils, (D) Treg.
