# Supplementary material for: Non‐Invasive Assessment of Complete Regression in Endometrial Cancer Patients Undergoing Fertility Preservation Using MRI‐Based Radiomics and Immune Heterogeneity
Source: MedComm (2020). 2026 Mar 4;7(3):e70666. doi: 10.1002/mco2.70666 (PMC12960057; doi:10.1002/mco2.70666)
Supplement: Supplementary file 2 — Table S1: Cell marker for scRNA clustering. [file MCO2-7-e70666-s002.docx]

Table S1 Cell marker for scRNA clustering

| Cell type | Marker |
| --- | --- |
| Epithelial cell | CDH1; CLDN4; EPCAM; KLF5; KRT14; KRT19 |
| Fibroblast | ACTA2; COL1A1; COL1A2; COL3A1; DCN; LUM |
| Macrophage | CD163; CD68; CSF1R; IL1B |
| NK cell | GNLY; NCAM1; NKG7 |
| T cell | CD3D; CD3E; CD4; CD8A; IL7R; PTPRC |
| Smooth muscle cell | ACTA2; RGS5; TAGLN |
| Proliferative cell | CENPF; MKI67; STMN1; TOP2A |
| Plasma cell | CD27; IGHG1; IGLL1; JCHAIN; CD38 |
